# Supplementary material for: RNF213 Loss-of-Function Promotes Angiogenesis of Cerebral Microvascular Endothelial Cells in a Cellular State Dependent Manner
Source: Cells. 2022 Dec 24;12(1):78. doi: 10.3390/cells12010078 (PMC9818782; doi:10.3390/cells12010078)
Supplement: Supplementary file 1 [file cells-12-00078-s001.zip › SupplementaryFigures_RNF213angio_revision (optimis¿a).pdf]

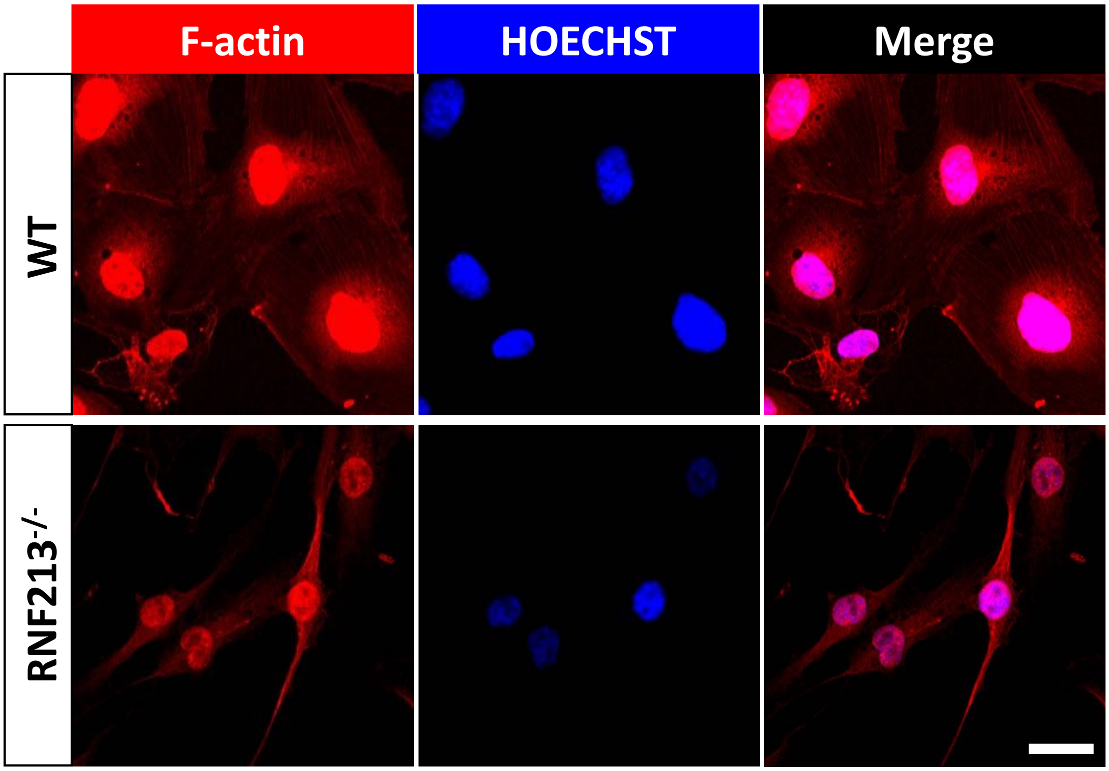

Figure S1. Morphology changes of hCMEC/D3 deficient in RNF213. Analysis of cells morphology using F-actin (red) immunostaining to visualise the cytoskeleton. Scale bar = 25 μm.

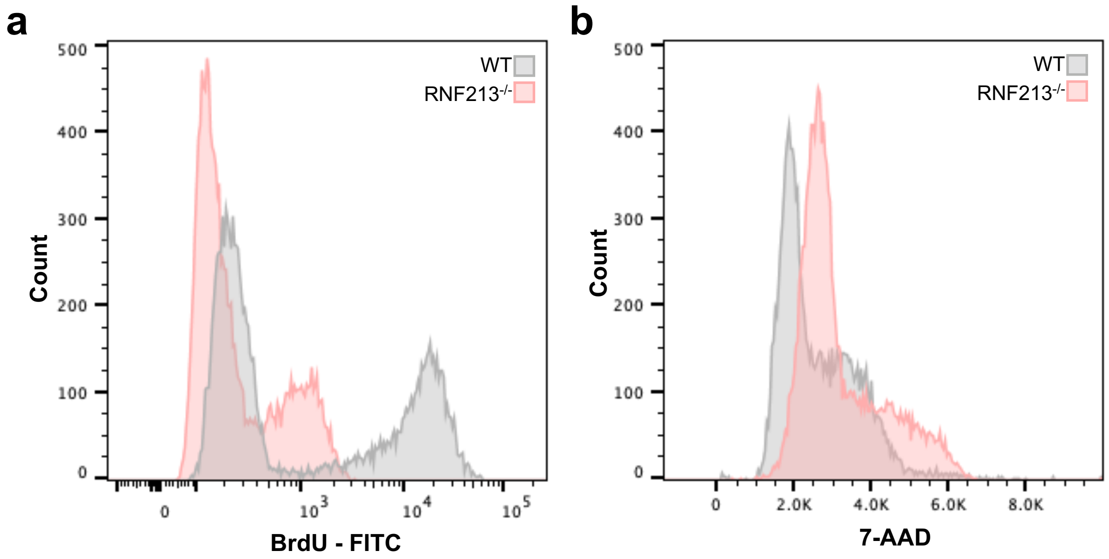

Figure S2. Histogram representation of the cell-cycle phase detection analysis. (**a**) Histogram of proliferative hCMEC/D3-WT and hCMEC/D3-RNF213$^{-/-}$ stained with BrdU – FITC. (**b**) Histogram of proliferative hCMEC/D3-WT and hCMEC/D3-RNF213$^{-/-}$ stained with 7-AAD.

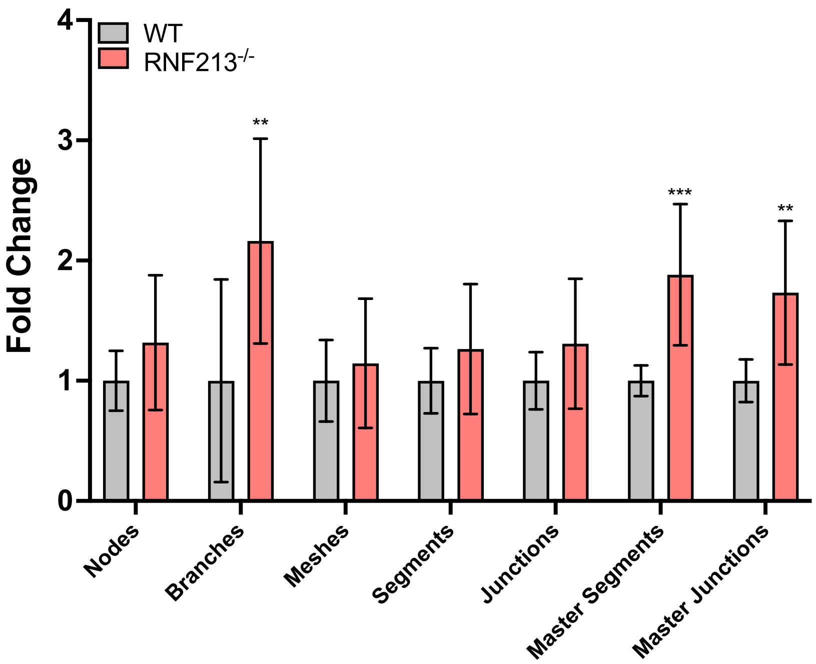

Figure S3. Evaluation of RNF213 knockout on angiogenic parameters of hCMEC/D3. The angiogenesis analyser plugin on ImageJ was used for the analysis. For each parameter, measurements were reported in fold change relative to WT control. $n = 10$. ** $p < 0.01$ and *** $p < 0.001$.

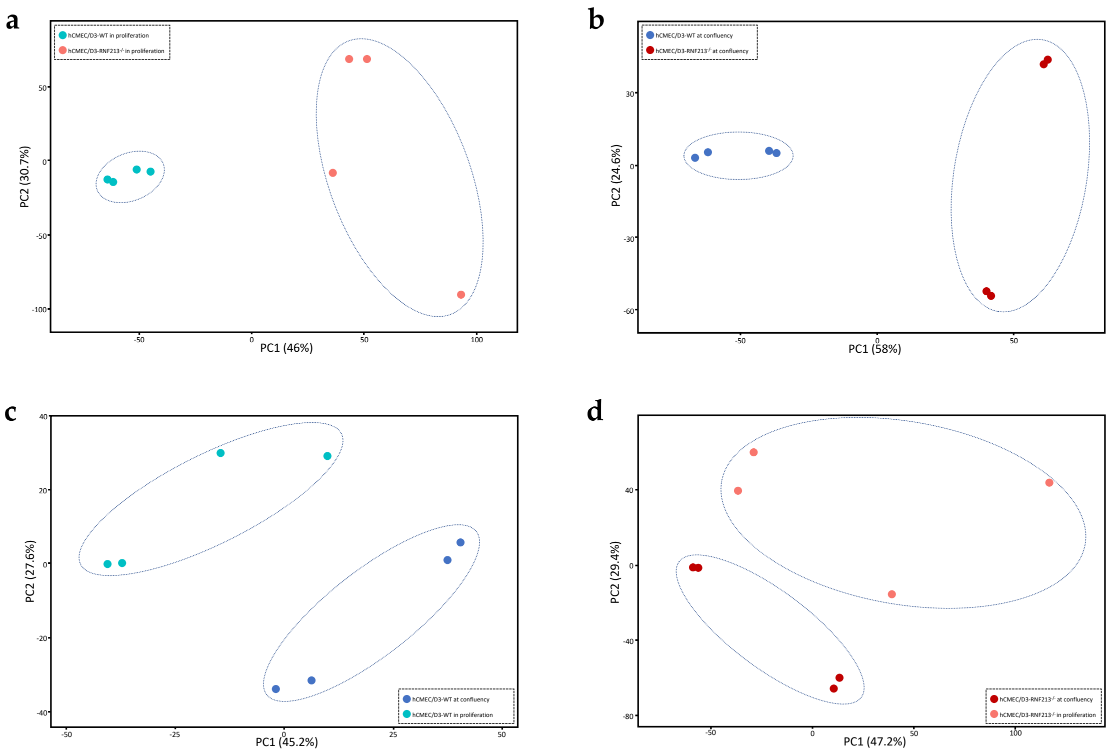

Figure S4. PCA analysis biplot showing a distinct gene expression clustering for hCMEC/D3-WT and -RNF213$^{-/-}$ cultured in a proliferative state (**a**) or at confluency (**b**) and for the passage from proliferative to confluent hCMEC/D3-WT cells (**c**) or hCMEC/D3-RNF213$^{-/-}$ cells (**d**). *n* = 4/groups.

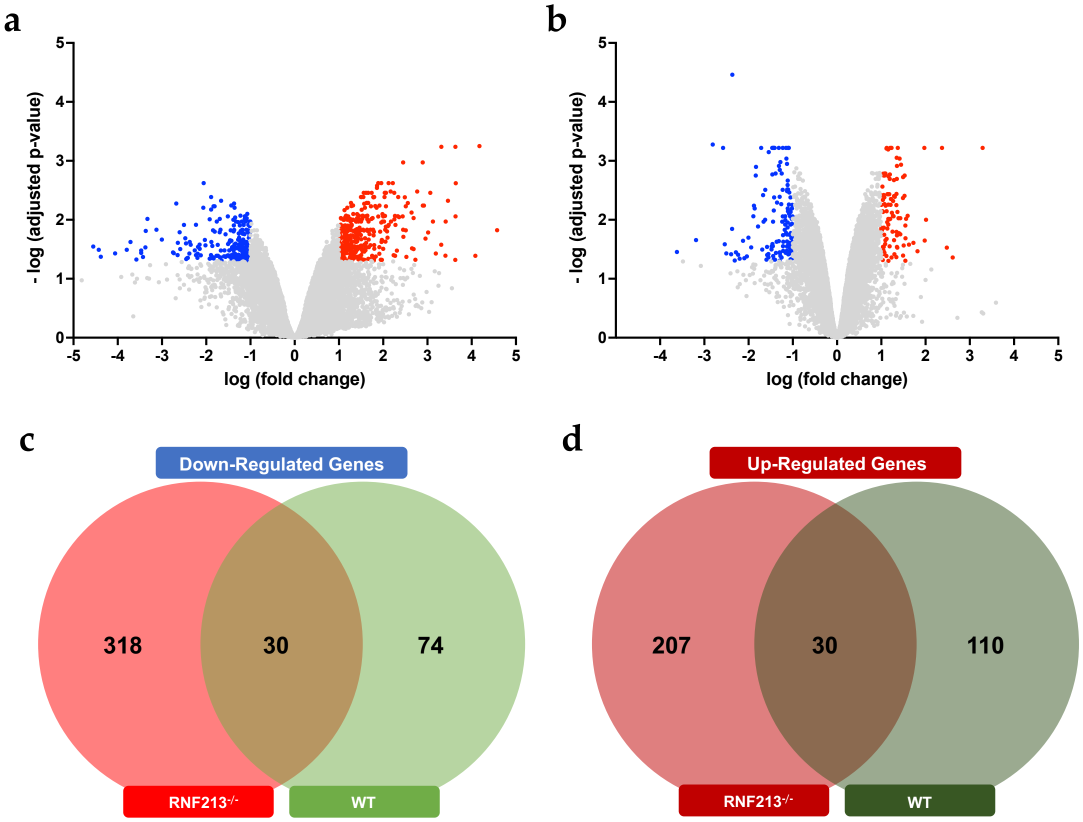

Figure S5. Modulation of genes in wild-type and RNF213 knockout hCMEC/D3 when cultured from proliferative to confluent state. Volcano plots showing the DEGs in RNF213 knockout endothelial cells (**a)** and wild-type cells (**b**) in function of the cellular state. Venn diagrams of significantly down-regulated (**c**) and up-regulated genes (**d**) in RNF213 knockout or wild-type endothelial cells. n = 4/groups.

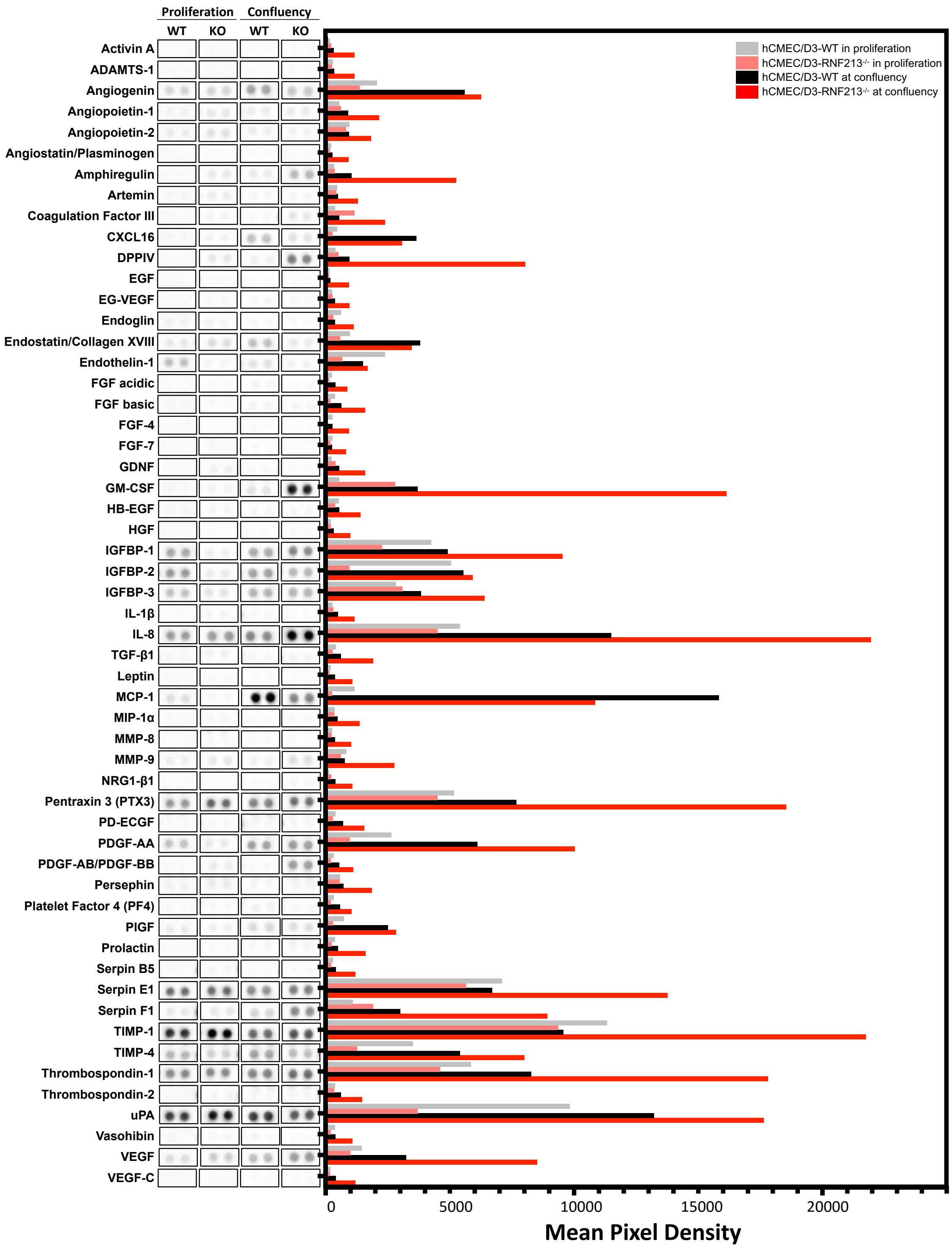

Figure S6. Secreted angiogenic factors from hCMEC/D3 cultured in proliferation and at confluency. Analysis of all secreted angiogenic proteins by hCMEC/D3-WT and hCMEC/D3-RNF213$^{-/-}$ cultured at a different state using the human angiogenesis proteome profiler. Mean pixel density was normalised to the number of cells present in the well at the moment of the supernatant recovery. $n = 2$/groups.

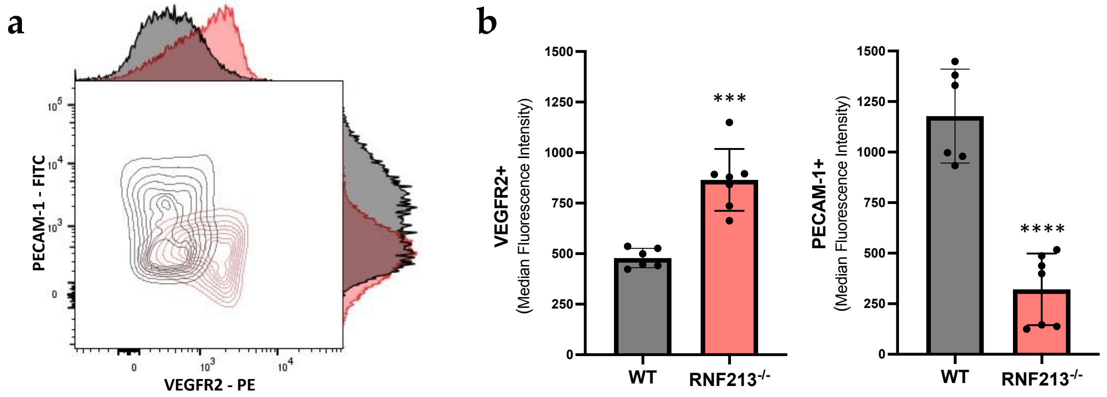

Figure S7. Plasma membrane expression of VEGFR2 and PECAM-1. (**a**) Flow cytometry analysis of VEGFR2/PECAM-1 expressed at the cell surface of hCMEC/D3-WT (black) and hCMEC/D3-RNF213$^{-/-}$ (red); (**b**) VEGFR2-positive and PECAM-1-positive cells' median fluorescence intensities of hCMEC/D3-WT and hCMEC/D3-RNF213$^{-/-}$. n = 6-7. *** $p < 0.001$ and **** $p < 0.0001$.

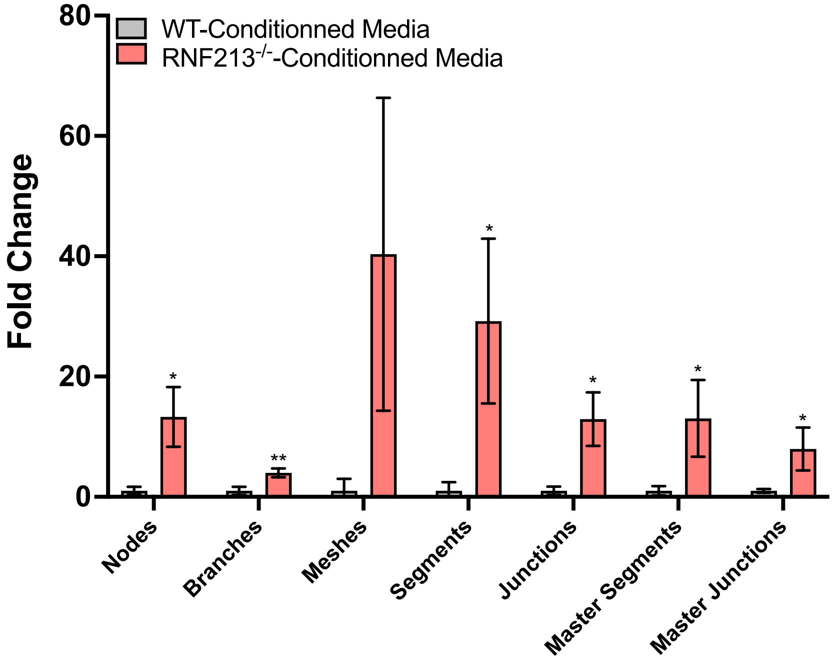

Figure S8. Effect of conditioned media derived from WT and RNF213 knockout hCMEC/D3 on angiogenic parameters. The angiogenesis analyser plugin on ImageJ was used for the analysis. For each parameter, measurements were reported in fold change relative to WT control. $n = 10$. * $p < 0.05$.

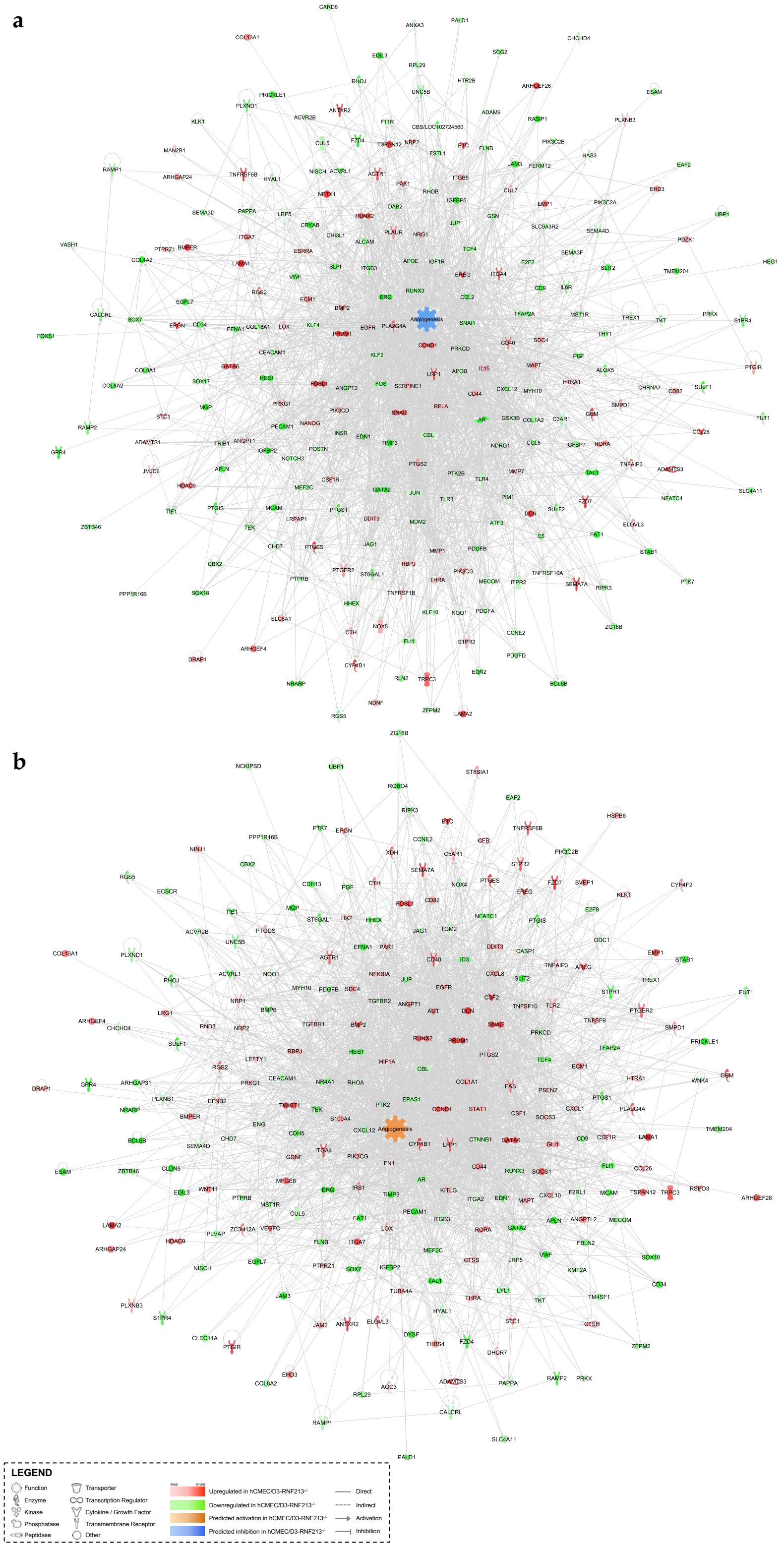

Figure S9. Ingenuity Pathway Analysis generated interactome of differently expressed genes linked with to angiogenesis. The interactomes highpoint DEGs directly and indirectly involved with angiogenesis in hCMEC/D3 deficient in RNF213 cultured in a proliferative (**a**) and a confluent (**b**) state.

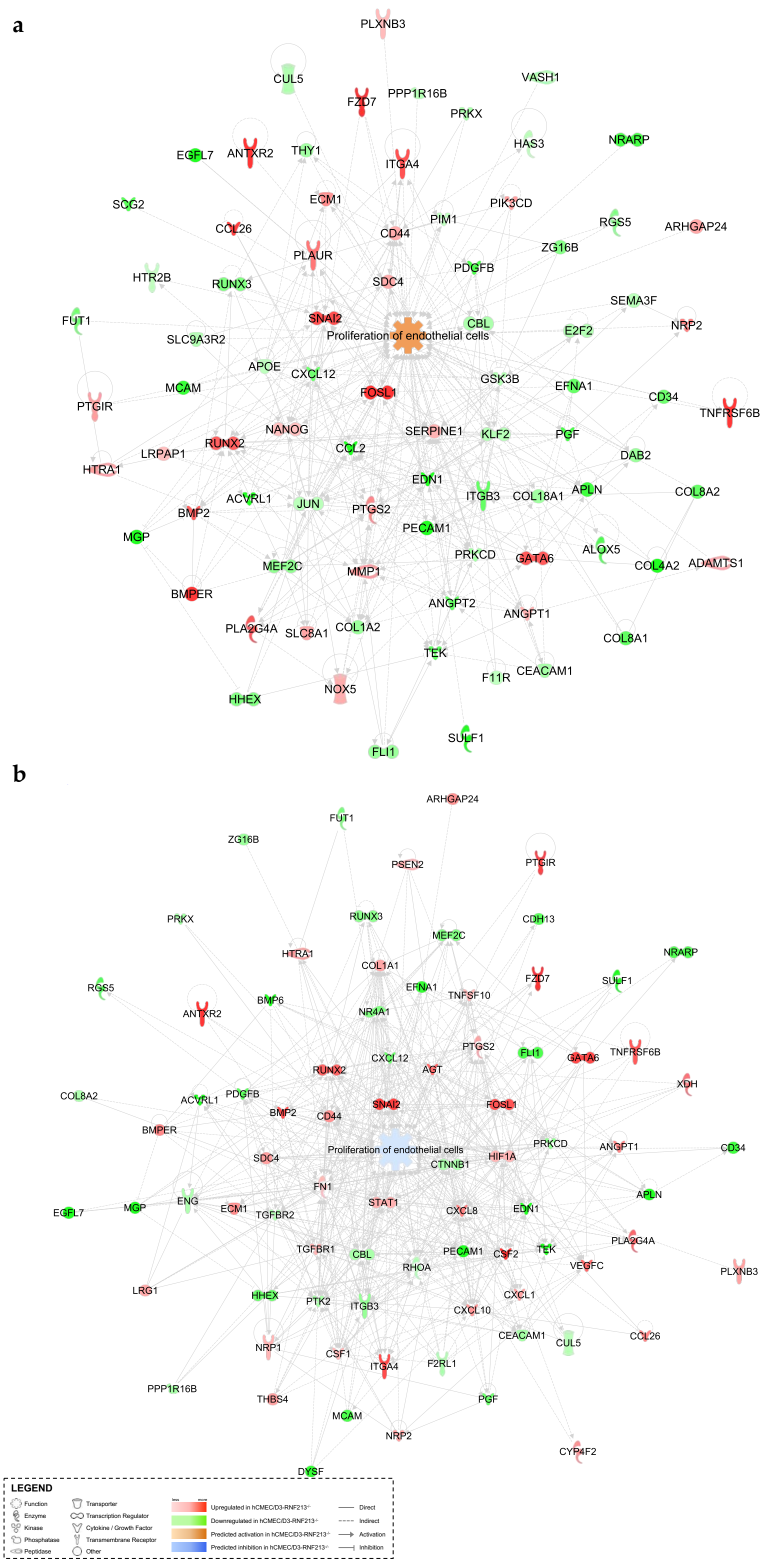

Figure S10. Ingenuity Pathway Analysis generated interactome of differently expressed genes linked with to endothelial cell proliferation. The interactomes highpoint DEGs directly and indirectly involved with the proliferation in endothelial cells in hCMEC/D3 deficient in RNF213 cultured in a proliferative (**a**) and a confluent (**b**) state.

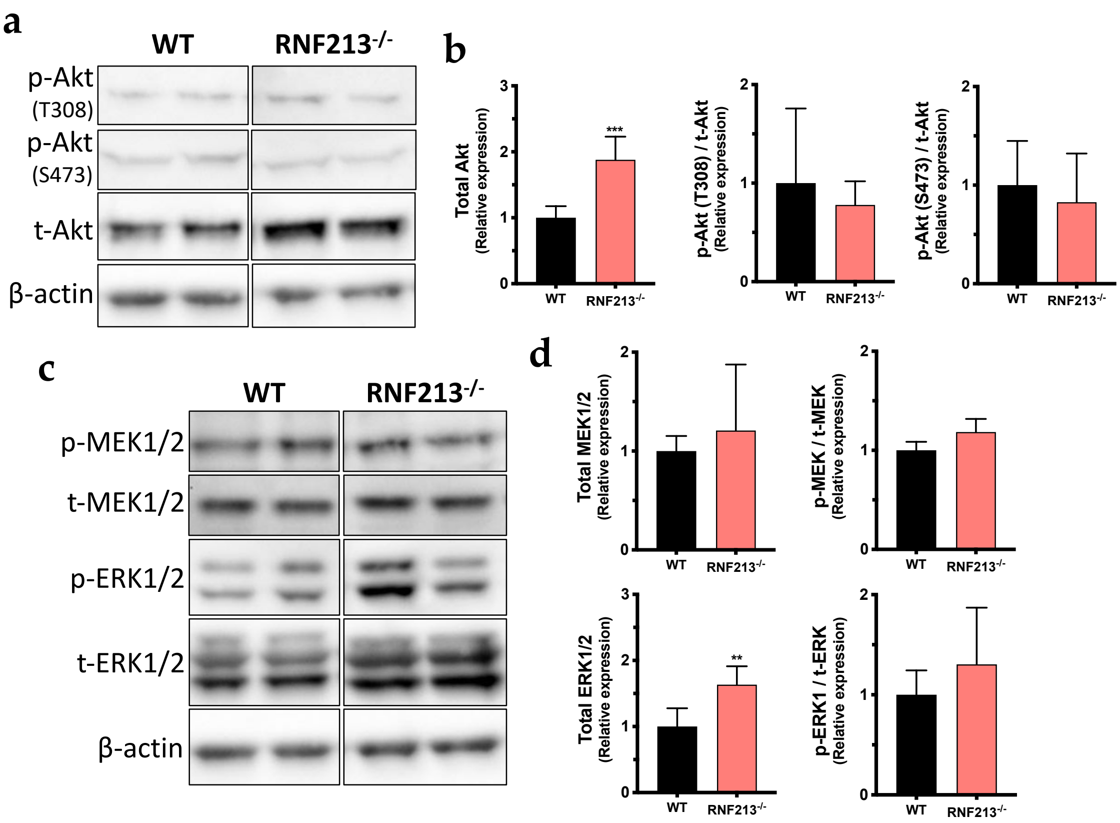

Figure S11. Expression and phosphorylation levels of the Akt kinase and MAP kinases in hCMEC/D3-WT and hCMEC/D3-RNF213$^{-/-}$ cultured in a proliferative state. (**a**) Western blot analysis of the Akt kinase and the two phosphorylated forms (T308 and S473) in both hCMEC/D3 groups. B-actin was used as a loading control. (**b**) Densitometry analysis of the total Akt and the phosphorylation levels. Total Akt levels are 11ormalized to β-actin and shown to the relative intensity of the WT control. Both phosphorylated forms of Akt were 11ormalized to total Akt. (**c**) Western blot analysis of the MAP kinases MEK1/2 and ERK1/2 and their phosphorylated forms in both hCMEC/D3 groups. (**d**) Densitometry analysis of the total MEK1/2 and ERK1/2 expression and their phosphorylation levels. Normalised total MEK1/2 and ERK1/2 protein levels are shown as fold expression, relative to the control. Phosphorylated forms of MEK1/2 and ERK1/2 were 11ormalized to total MEK1/2 and ERK1/2, respectively. n = 6-7. ** $p < 0.01$ and *** $p < 0.001$.

a

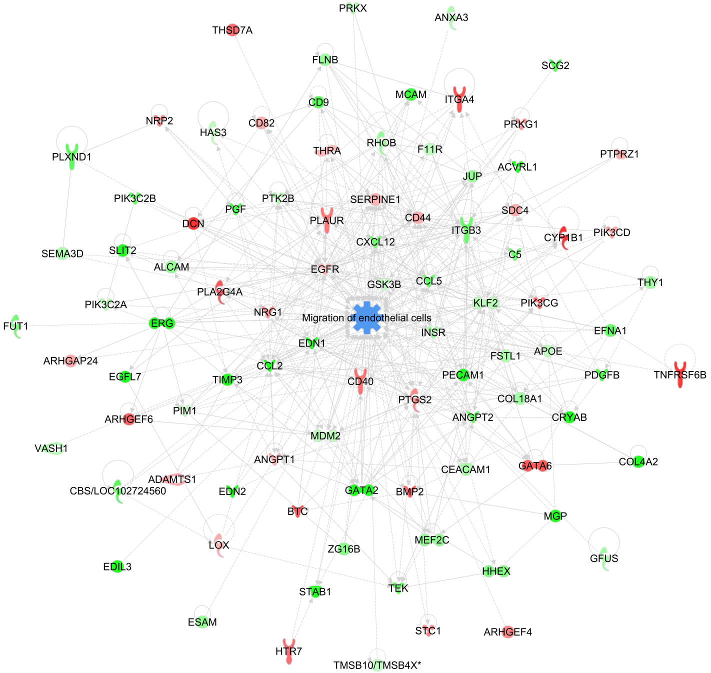

b

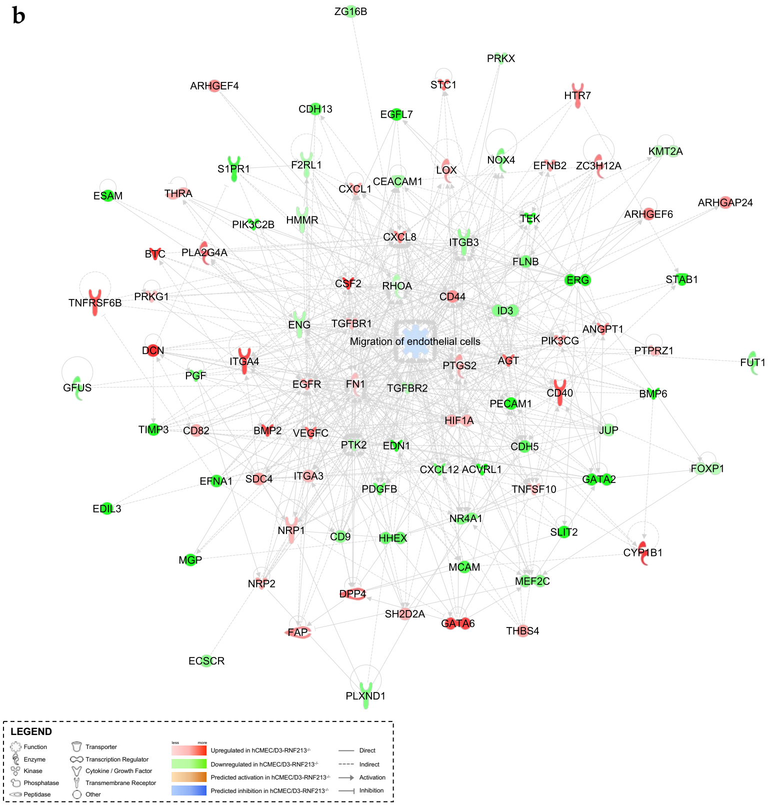

Figure S12. Ingenuity Pathway Analysis generated interactome of differently expressed genes linked with to endothelial cell migration. The interactomes highpoint DEGs directly and indirectly involved with the migration in endothelial cells in hCMEC/D3 deficient in RNF213 cultured in a proliferative (**a**) and a confluent (**b**) state.

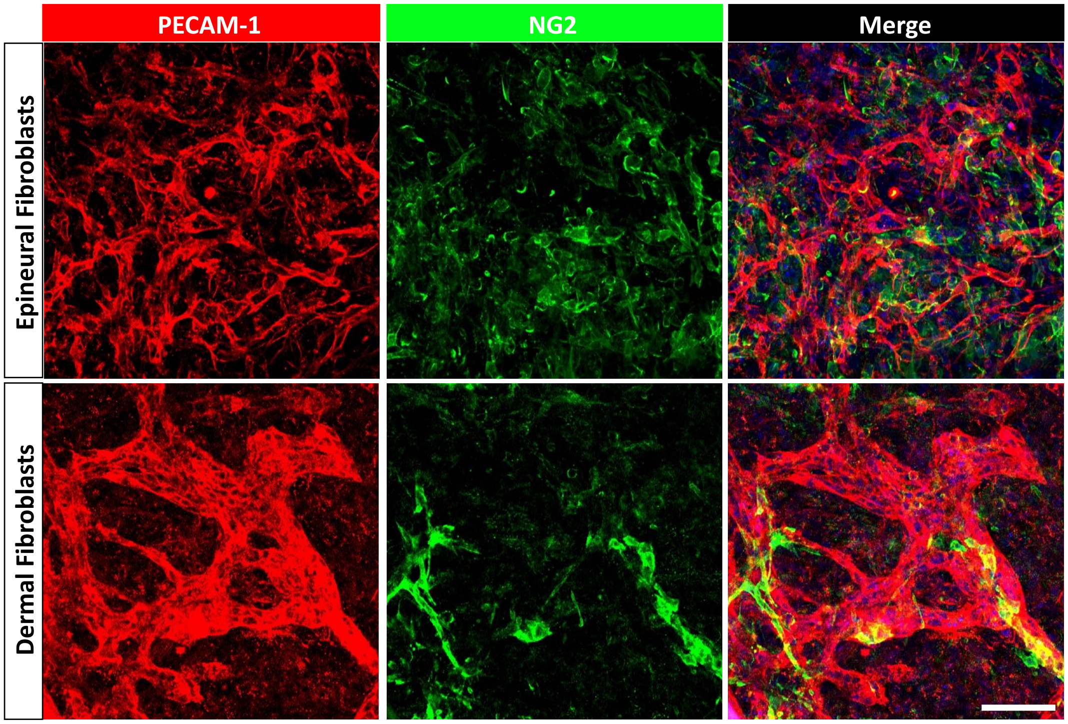

Figure S13. Microcapillaries formation in self-assembled constructs. Epineural fibroblasts and dermal fibroblasts were compared as a stromal support for capillary-like formation. 3D microvascular networks were visualised by immunofluorescence staining for PECAM-1 (red) and NG2 (green) that were imaged by confocal microscopy. Nuclei were stained with Hoechst (blue). Scale bar = 200 μm.
